# Family Caregiver Role in the Long-Term Services and Supports of Individuals With Dementia Over Time

**DOI:** 10.1093/geroni/igaa057.1571

**Published:** 2020-12-16

**Authors:** Jennifer Reckrey, Evan Bollens-Lund, Mohammed Husain, Katherine Ornstein, Amy Kelley

**Affiliations:** Icahn School of Medicine at Mount Sinai, New York, New York, United States

## Abstract

Family caregivers of persons living with dementia (PLWD) provide disproportionately high levels of care over a long and variable disease course, yet an understanding of the trajectory of care hours provided over time and the contributions of individual family members to overall care is lacking. This study used longitudinal data from the nationally representative Health and Retirement Study in order to compare the hours of care that spouses, children, and other family caregivers provide to those with and without dementia. During the last 10 years of life, family caregivers of PLWD provided nearly three times as many total care hours as compared to others (7,447 vs. 2,653 total hours). While care hours provided to PLWD increased steadily in each of the last 10 years of life (going from 4 hours/week 10 years before death to 33 hours/week the year before death, average annual increase 27%), care hours provided to others remained low and then nearly tripled in the last year of life to 22 hours/week on average. Adult children of PLWD provided 50% of total care hours, while adult children of others provided 41% of care hours. This study provides important insight into the high levels of year-over-year caregiving provided to PLWD by their family caregivers in general and by adult children in particular. Policies to support these caregivers must shift from short-term, episodic support to sustained assistance in acknowledgment of the key role family caregivers play in the long-term services and supports of PLWD.

